# Coordination chemistry of polynitriles. Part 9. Deca­cyano­ferrocene revisited: crystal and mol­ecular structure of *cis*-[{C_5_(CN)_5_}_2_(MeCN)_4_Fe]

**DOI:** 10.1107/S2053229622000365

**Published:** 2022-01-21

**Authors:** Karlheinz Sünkel, Tobias Blockhaus

**Affiliations:** a Ludwig-Maximilians-Universität München, Department Chemie, Butenandtstrasse 9, 81377 Munich, Germany

**Keywords:** ferrocene, penta­cyano­cyclo­penta­dienide, π–π inter­actions, weak inter­actions, deca­cyano­ferrocene, crystal structure

## Abstract

The reaction of Ag[C_5_(CN)_5_] with anhydrous FeCl_2_ in aceto­nitrile leads to colourless crystals of tetra­kis­(aceto­nitrile-κ*N*)bis­(penta­cyano­cyclo­penta­dienido-κ*N*)iron(II) aceto­nitrile 1.8-solvate, [Fe(C_10_N_5_)_2_(CH_3_CN)_4_]·1.8CH_3_CN or *cis*-[{C_5_(CN)_5_}_2_(MeCN)_4_Fe]·1.8MeCN. The com­pound crystallizes in the triclinic space group *P*  as monomers, which exhibit weak C—H⋯N and π–π inter­actions. The crystals contain *ca* 20% solvent-accessible voids, which are nearly com­pletely filled by two MeCN mol­ecules.

## Abstract 2

The crystal structure determination of the enigmatic ‘deca­cyano­ferrocene’ identifies this com­pound as *cis*-[{C_5_(CN)_5_}_2_(MeCN)_4_Fe].

## Introduction

The term ‘deca­cyano­ferrocene’ appeared first in a publication about ‘diazo­tetra­cyano­cyclo­penta­diene’ (Webster, 1966 ▸) and later in two US patents by the same author (Webster, 1970 ▸, 1974 ▸). It was used for the reaction product from silver penta­cyano­cyclo­penta­dienide and FeCl_2_ in aceto­nitrile, which led to ‘light-green crystals of deca­cyano­ferrocene’, which were char­acterized, after drying at 112 °C under vacuum, by ele­mental analysis and IR and UV spectroscopy as ‘C_20_N_10_Fe·*x*H_2_O’. No indication or proof was given for the formulation as a ‘ferrocene’. A couple of years later, a different research group repeated the experiment and described the primary product as ‘white crystals’ (Christopher & Venanzi, 1973 ▸). Drying of the crystals at room temperature *in vacuo* produced a white solid that still, according to its IR spectrum, contained aceto­nitrile. Further drying at 110 °C *in vacuo* produced a pale-yellow–green product, which analyzed as ‘C_20_N_10_Fe·*x*H_2_O’ and was further characterized by IR spectroscopy and magnetic and conductivity measurements. In the absence of a crystal structure determination, these authors postulated a ‘polymeric structure in which the iron is in an approximately octa­hedral environment’, in which ‘each PP group bridges three iron atoms’. Within the last 15 years, the coordination chemistry of the penta­cyano­cyclo­penta­dienide anion has been studied intensively by us and others (Sünkel & Reimann, 2013 ▸; Sünkel & Nimax, 2018 ▸; Nimax *et al.*, 2018 ▸; Blockhaus & Sünkel, 2021 ▸; Bacsa *et al.*, 2011 ▸; Less *et al.*, 2013 ▸). These studies showed that [C_5_(CN)_5_]^−^ could behave either as a noncoordinating anion or use one to its five cyano groups for coordination, sometimes even in a bridging μ_2_-κ^1^:κ^1^ fashion. We had also treated FeCl_2_ with Ag[C_5_(CN)_5_] in methanol. Recrystallization from MeOH gave crystals of *trans*-[{C_5_(CN)_5_}_2_Fe(H_2_O)_4_], in which both anions used only one cyano function each for coordination to iron in a mononuclear com­pound (Sünkel *et al.*, 2019 ▸). Individual mol­ecules were connected *via* hydrogen bridges into a three-dimensional network. Since all the above-mentioned reports described the formation of (either ‘light green’ or ‘white’) crystals as the primary product of the reaction in aceto­nitrile, we decided to repeat this reaction and to study the crystals. 
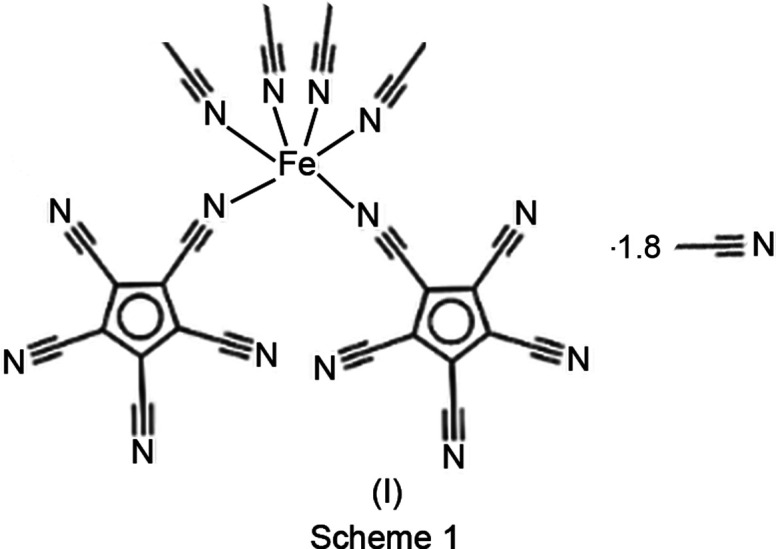


## Experimental

### Synthesis and crystallization

The title com­pound, tetra­kis­(aceto­nitrile-κ*N*)bis­(penta­cyano­cyclo­penta­dienido-κ*N*)iron(II) aceto­nitrile disolvate, (I) (Scheme 1), was prepared as described in the literature (Webster, 1966 ▸; Christopher & Venanzi, 1973 ▸). Recrystallization of the crude product by slow evaporation of an aceto­nitrile solution under an argon atmosphere gave colourless crystals suitable for X-ray diffraction analysis. Heating the crystals at 110 °C *in vacuo* for several hours left an amorphous powder. All attempts to obtain crystals of this product by dissolution in a noncoordinating solvent met with failure.

### Refinement

**Figure 1:**
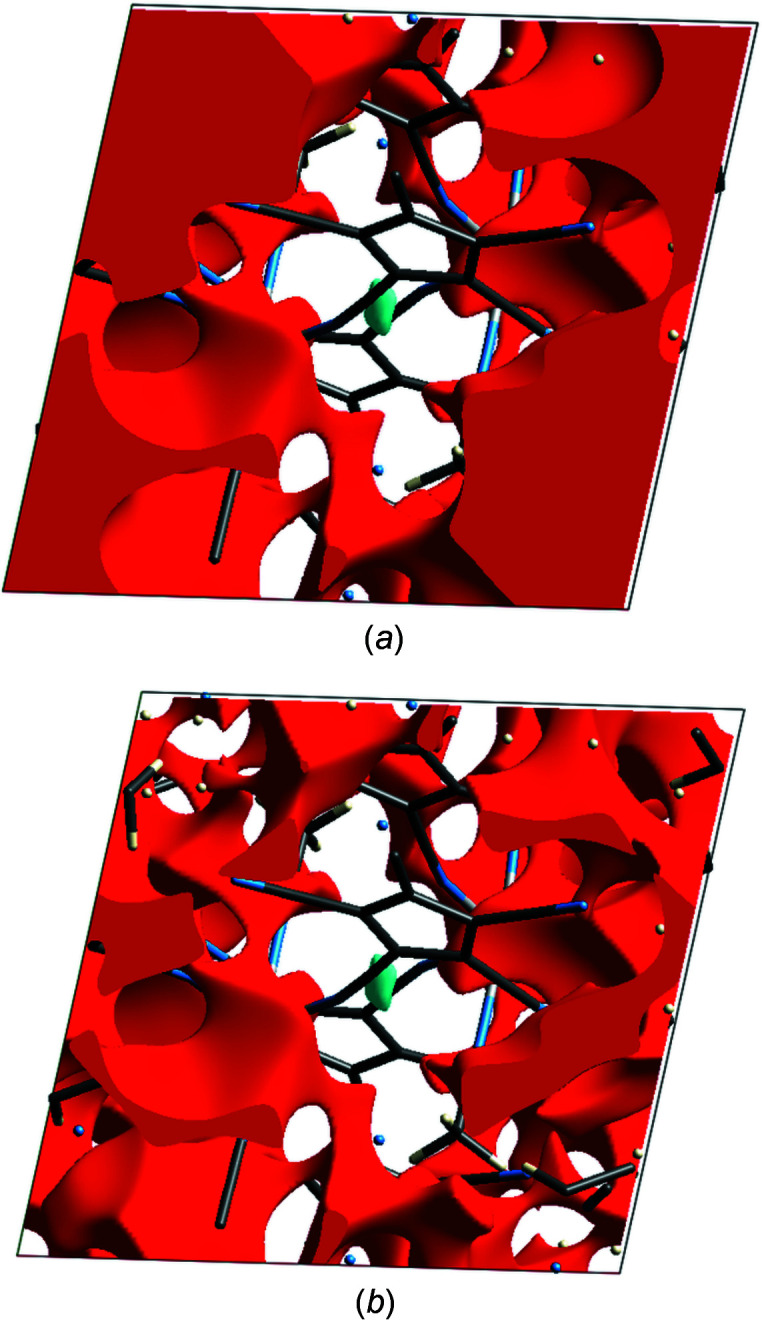
Crystal void plots (0.002 a.u. isosurface) of the crystal structure (*a*) without and (*b*) including the MeCN lattice mol­ecules.

The structure refinement showed, besides the mol­ecular unit, two lattice aceto­nitrile (MeCN) mol­ecules, which were both disordered. The disorder of one MeCN mol­ecule could be resolved with the help of restraints into two positions in relative 80:20 occupancies. The disorder of the second molecule, however, could not be resolved. Due to some unfavourable close contacts with the ‘minor’ molecule, the site-occupancy factor (s.o.f.) of the second molecule was reduced to 0.8 anyway. After inclusion of these MeCN mol­ecules, *PLATON* (Spek, 2020 ▸) analysis showed no more solvent-accessible voids. The results of the refinement using this model are shown in the second column of Table 1 ▸. As the *PLATON* analysis of the structure without the lattice aceto­nitrile mol­ecules showed 20% solvent-accessible voids (for a ‘cavity plot’, see Fig.  S1 of the supporting information), a refinement using the SQUEEZE routine (Spek, 2015 ▸) was tried. The results of this refinement are shown in the third column of Table 1 ▸. As can be seen, the SQUEEZE refinement led to slightly better *R* values. To obtain further insight into the importance of crystal voids in this structure, the ‘un-SQUEEZEd’ CIF file was examined using the program *CrystalExplorer* (Version 21.5), using the subroutine ‘void’ (Turner *et al.*, 2011 ▸), both without and with the aceto­nitrile mol­ecules. Fig.  1 ▸(*a*) shows the void plot obtained without the MeCN mol­ecules, while Fig.  1 ▸(*b*) shows the same plot when the MeCN mol­ecules were included (0.002 a.u. isosurfaces; for the results of the corresponding calculations using 0.0003 a.u. isosurfaces, see Fig.  S2 in the supporting information). Table 2 ▸ summarizes the results of the void-space calculations using *PLATON* and *CrystalExplorer*.

As can be seen, the results obtained with *PLATON* (excluding the MeCN solvents) are inter­mediate between the *CrystalExplorer* results with the two different isosurfaces, which is rather unusual (Turner *et al.*, 2011 ▸). After inclusion of the MeCN mol­ecules, the *PLATON* results and the *CrystalExplorer* results for a 0.0003 a.u. surface are nearly identical, and show that there are no permanent voids left after inclusion of the MeCN mol­ecules. In view of this, together with the probable involvement of the lattice MeCN mol­ecules in C—H⋯N hydrogen bonds, the SQUEEZEd structure was not examined further.

## Results and discussion

**Figure 2:**
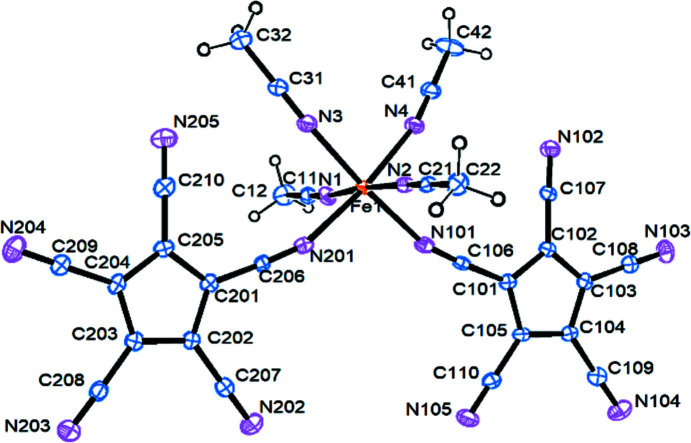
Displacement ellipsoid plot (30% probability level) of (I). The lattice MeCN mol­ecules are not shown.

The title com­pound crystallizes in the triclinic space group *P* 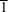 with one mol­ecule in the asymmetric unit. The Fe^II^ ion coordinates to two *cis*-oriented penta­cyano­cyclo­penta­dienyl anions *via* one nitrile function each, and additionally to four aceto­nitrile mol­ecules (Fig.  2 ▸).

The two cyclo­penta­dienyl rings are coplanar [inter­planar angle = 0.8 (2)°; the average distance of atoms C201–C205 from the best plane through C101–C105 is 0.044 ± 0.02 Å]. The bond lengths from the Fe atom to the [C_5_(CN)_5_] N atoms are significantly (>10σ) longer [average 2.176 (3) Å] than to the aceto­nitrile N atoms [average 2.140 (4) Å], with the bond angles at the coordinating atoms N101 and N201 close to being linear (average 161.9°). Further important bond parameters can be found in Table 3 ▸.

**Figure 3:**
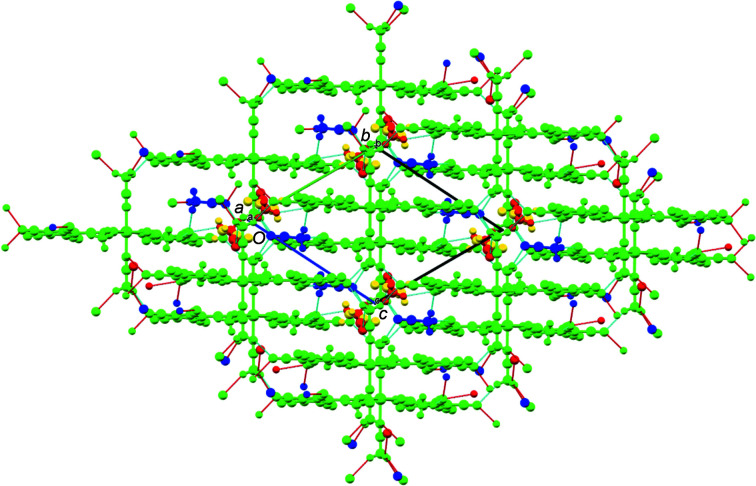
Packing plot (*Mercury*; Macrae *et al.*, 2020 ▸), viewed along the crystallographic *a* axis. The colour coding green/blue/yellow/red corresponds to the symmetry equivalents, as defined by *Mercury*. Red and blue lines show the hydrogen bonds according to Table 4 ▸.

Weak inter­actions with the contents of the voids contribute to the stability of the crystal lattice (Ghosh *et al.*, 2019 ▸; Wang *et al.*, 2020 ▸) and so a closer inspection of the packing plots seemed appropriate (Fig.  3 ▸).

A packing plot viewed down the crystallographic *a* axis shows ‘layers’ of cyclo­penta­dienyl rings oriented parallel to the *bc* diagonal and orthogonal to the plane of projection. These layers contain also one of the lattice MeCN mol­ecules (dark blue in Fig.  3 ▸). Individual mol­ecules are connected *via* C—H⋯N hydrogen bonds in the *b* and *c* directions using methyl groups C12 and C22 of the coordinated aceto­nitrile mol­ecules (both in *cis* positions relative to the coordinated anions) as donors and the penta­cyano­cyclo­penta­dienide atoms N103 and N204 (in the 3-position relative to the coordinated cyano N atom), as well as the lattice MeCN atoms N5 and N6*B*, as acceptors. In addition, there is also a hydrogen bond between the lattice MeCN group C52 and penta­cyano­cyclo­penta­dienide atom N102 (Table 4 ▸).

**Figure 4:**
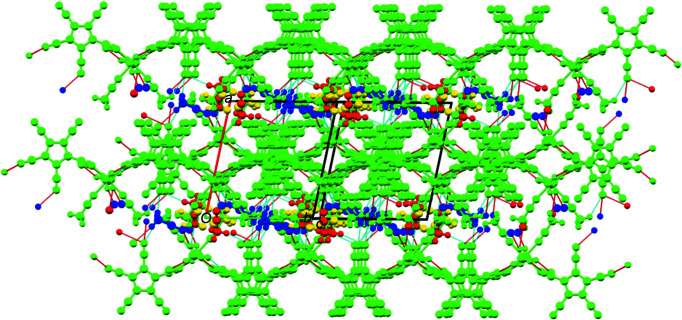
Packing plot (*Mercury*; Macrae *et al.*, 2020 ▸), viewed perpendicular to the *bc* plane. The colour coding is as in Fig.  3 ▸.

A possibly more important inter­molecular inter­action becomes visible in Fig.  4 ▸.

**Figure 5:**
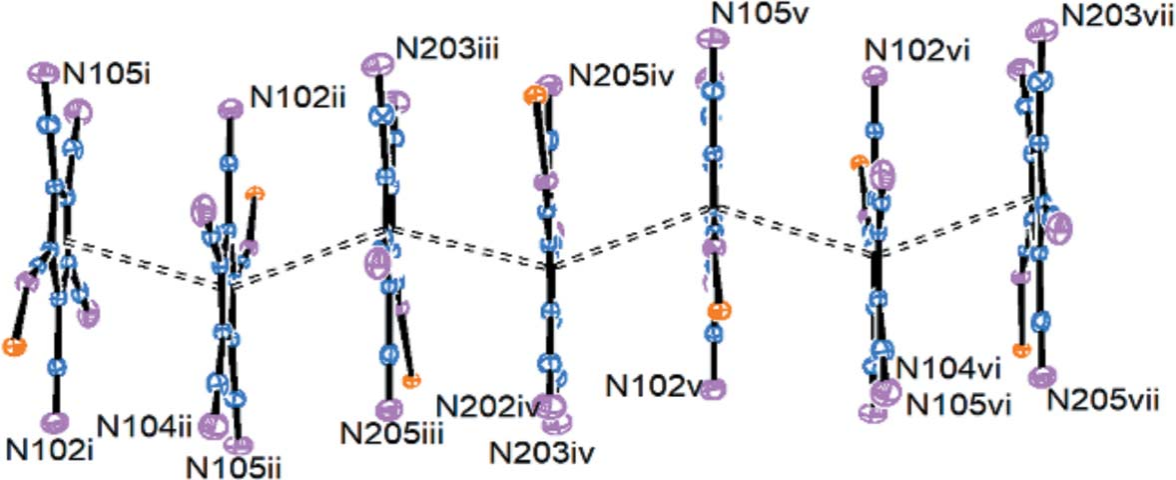
π-Stacking of the cyclo­penta­dienyl rings. [Symmetry codes: (i) *x*, *y* + 1, *z* − 1; (ii) −*x* + 1, −*y* + 1, −*z*; (iii) *x*, *y*, *z* − 1; (iv) −*x* + 1, −*y* + 1, −*z* + 1; (v) *x*, *y*, *z*; (vi) −*x* + 1, −*y*, −*z* + 1; (vii) *x*, *y* − 1, *z*.]

The penta­cyano­cyclo­penta­dienyl rings stack *via* π–π inter­actions (Carter-Fenk & Herbert, 2020 ▸; Thakuria *et al.*, 2019 ▸), with the ring planes at a typical distance of *ca* 3.36 Å. A closer look (Fig.  5 ▸) shows that the stack is formed by alternating pairs of inversion-related C101–C105 (symmetry codes i/ii and v/vi) and C201–C205 (iii/iv) rings. The dotted ‘bonds’ in Fig.  5 ▸ join the ring centroids at distances of 3.575 (i/ii and v/vi), 3.580 (ii/iii, iv/v and vi/vii) and 3.597 Å (iii/iv), and angles of 141.3 and 142.8°. This corresponds to a ‘ring slippage’ of *ca* 1.15 Å.

In order to gain further insight into the inter­actions at work, a Hirshfeld analysis was undertaken with the help of the program *CrystalExplorer* (Spackman *et al.*, 2021 ▸).

**Figure 6:**
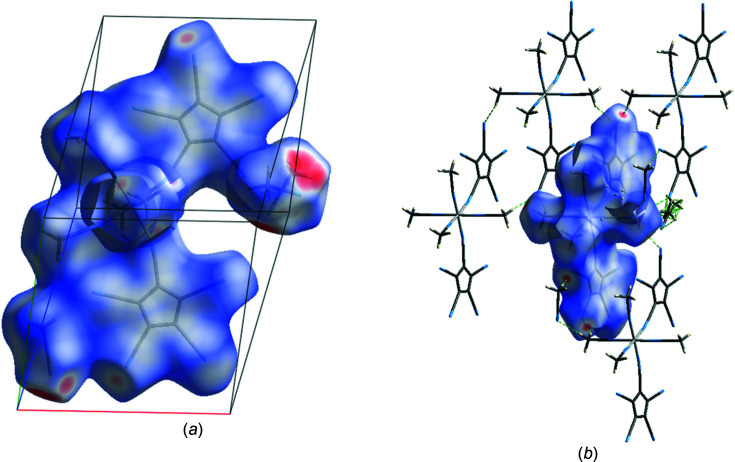
(*a*) Hirshfeld surface of the asymmetric unit of (I) using normalized contact distances (*d*_norm_) for colour coding. Red areas represent regions where the contact distances are significantly below the sum of the van der Waals radii. (*b*) Hirshfeld surface of one isolated metal com­plex, together with some close-by neighbours, indicating also the hydrogen bridges between them and the central fragment.

**Figure 7:**
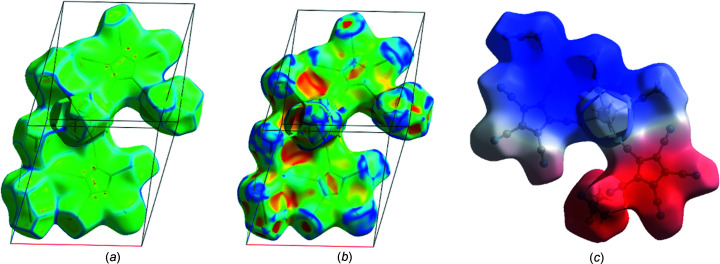
Hirshfeld surfaces displaying the properties (*a*) ‘curvedness’, (*b*) ‘shape index’ and (*c*) ‘electrostatic potential’.

Fig.  6 ▸(*a*) shows the Hirshfeld surface of the asymmetric unit, with the *d*_norm_ surface property (range −0.65 to 1.30). The strong involvement of the lattice MeCN mol­ecules in donor C—H⋯N (top right) and acceptor N⋯H—C (bottom left) inter­actions can be seen. Fig.  6 ▸(*b*) shows the Hirshfeld surface of an isolated com­plex fragment and its inter­actions with four further com­plex fragments and a few lattice MeCN mol­ecules. In Fig.  7 ▸, the same surface showing the properties ‘curvedness’, ‘shape index’ and ‘electrostatic potential’ is displayed.

Both the ‘curvedness’ and the ‘shape index’ plots show the importance of planar π-stacking for both cyclo­penta­dienyl rings (Spackman & Jayatilaka, 2009 ▸). Fig.  7 ▸(*c*) shows that the asymmetric unit contains both electropositive (blue) and electronegative (red) parts, together with small neutral (white) areas. Fig.  S3 (see supporting information) shows how in neighbouring mol­ecules the positive and negative parts approach each other.

**Figure 8:**
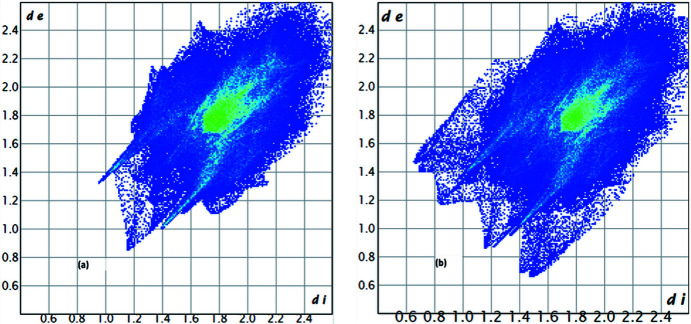
Fingerprint plots (*a*) for a Hirshfeld surface enclosing only the iron com­plex and (*b*) for the whole asymmetric unit.

The so-called ‘fingerprints’ are a graphical representation of all the inter­actions of atoms ‘inside’ and ‘outside’ the Hirshfeld surface (Spackman & McKinnon, 2002 ▸). Fig.  8 ▸(*a*) shows such a plot when the two lattice MeCN mol­ecules are left outside the Hirshfeld surface, while Fig.  8 ▸(*b*) represents such a plot when the com­plete asymmetric unit is inside the Hirshfeld surface. A plot showing the most important individual contributors is shown in Fig.  S4 (see supporting information). The bright-green spots at *ca* (1.8/1.8) Å in Fig.  8 ▸ correspond to π–π stacking inter­actions; inspection of Fig.  S4 shows that C⋯C inter­actions are responsible for *ca* 18% of all the inter­molecular inter­actions, while C—H⋯π inter­actions make up less than 7%. C—H⋯N contacts make up nearly 50% of the weak inter­actions.

**Figure 9:**
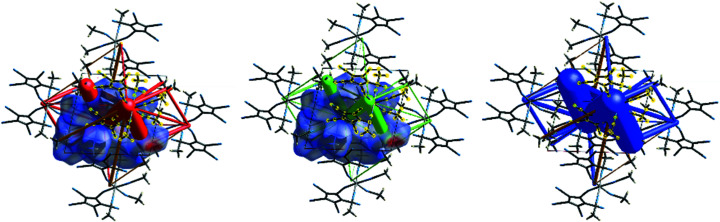
Energy frameworks, showing Coulombic (left) and dispersion (middle) terms, as well as total inter­action energies (right).

A last important point relates to the inter­action energies in the crystal. Fig.  S5 (see supporting information) shows that the inter­actions between the com­plex and the two unique MeCN solvent mol­ecules are relatively weak, with the repulsive terms dominating. The inter­actions between the asymmetric unit and four close neighbours are displayed in Fig.  S6. The energies range from −54 to −174 kJ mol^−1^. The strongest inter­action occurs for the closest approach of two inversion-related mol­ecules (magenta), with a clear dominance of the dispersion term. Another method for graphically representing these inter­actions is through the use of ‘energy frameworks’ (Turner *et al.*, 2015 ▸), which are displayed in Fig.  9 ▸.

## Conclusion

**Figure 10:**
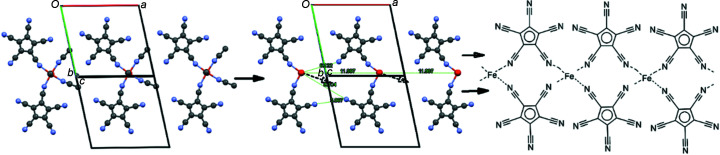
Suggested transformation from the title com­pound to a polymeric structure: (left) three mol­ecules along the *a* direction; (middle) the same part of the structure with the MeCN mol­ecules omitted; (right) sketch of the suggested polymer (without the bridging water ligands).

The primary reaction product from FeCl_2_ and Ag[C_5_(CN)_5_] in aceto­nitrile is neither a ‘ferrocene’ nor a coordination polymer. The structure determination presented here shows a mononuclear octa­hedral coordination com­pound with two *cis*-oriented monodentate penta­cyano­cyclo­penta­dienide anions and four aceto­nitrile ligands. The individual mol­ecules inter­act in the lattice *via* weak C—H⋯N hydrogen bonds and displaced parallel cyclo­penta­dienyl π-systems. In the absence of any crystals it is difficult to speculate about the structure of the com­pound ‘Fe[C_5_(CN)_5_]_2_·*x*H_2_O’ described over 50 years ago. However, one could imagine that after removal of all the aceto­nitrile mol­ecules, the remaining fragments approach each other parallel to the *bc* plane and form ‘ribbons’ of Fe[C_5_(CN)_5_]_4/2_ with the anions using two of their cyano groups, similar to the structure of Ca[C_5_(CN)_4_H]_2_·4H_2_O (Sünkel & Nimax, 2018 ▸). In contrast to the Ca com­pound, the Fe com­pound would have to be octa­hedrally coordinated with two additional bridging H_2_O ligands (Fig.  10 ▸).

## Supplementary Material

Crystal structure: contains datablock(s) I, global. DOI: 10.1107/S2053229622000365/eq3004sup1.cif

Structure factors: contains datablock(s) I. DOI: 10.1107/S2053229622000365/eq3004Isup2.hkl

Additional figures. DOI: 10.1107/S2053229622000365/eq3004sup3.pdf

CCDC reference: 2141277

## Figures and Tables

**Table 1:** Experimental details

	With localized solvent	With SQUEEZE
Crystal data		
Molecular formula	[Fe(C_10_N_5_)_2_(C_2_H_3_N)_4_]·1.8C_2_H_3_N	
Chemical formula	C_31.6_H_17.4_FeN_15.8_	C_28_H_12_FeN_14_
*M*_r_	674.29	600.37
Crystal system, space group	Triclinic, *P*\overline{1}	
Temperature (K)	109	
*a*, *b*, *c* (Å)	11.9972 (7), 12.8711 (7), 13.0907 (8)	
α, β, γ (°)	62.528 (2), 82.929 (2), 77.210 (2)	
*V* (Å^3^)	1748.47 (18)	
*Z*	2	
Radiation type	Mo *K*α	
μ (mm^−1^)	0.48	0.47
Crystal size (mm)	0.05 × 0.04 × 0.03	

Data collection		
Diffractometer	Bruker D8 Venture	
Absorption correction	Multi-scan (*SADABS*; Krause *et al.*, 2015 ▸)	
*T*_min_, *T*_max_	0.616, 0.745	
No. of measured, independent and observed [*I* > 2σ(*I*)] reflections	17051, 7081, 5145	17056, 7083, 5145
*R*_int_	0.042	
(sin θ/λ)_max_ (Å^−1^)	0.626	

Refinement		
*R*[*F* ^2^ > 2σ(*F* ^2^)], *wR*(*F* ^2^), *S*	0.064, 0.163, 1.03	0.061, 0.141, 1.04
No. of reflections	7081	7083
No. of parameters	464	392
No. of restraints	3	0
H-atom treatment	H-atom parameters constrained	
Δρ_max_, Δρ_min_ (e Å^−3^)	0.81, −0.63	0.77, −0.64

Computer programs: *APEX2* (Bruker, 2011 ▸), *SAINT* (Bruker, 2011 ▸), *SHELXT2014* (Sheldrick, 2015*a* ▸), *SHELXL2018* (Sheldrick, 2015*b* ▸) and *Mercury* (Macrae *et al.*, 2020 ▸).

**Table 2:** Comparison of the void calculations using *PLATON* and *CrystalExplorer* (*CE*) SASA is solvent accessible surface area (Düren *et al.*, 2007 ▸).

	*PLATON* VOID	*PLATON* SASA	*CE* (0.002 a.u.)	*CE* (0.0003 a.u.)
Without MeCN				
Void volume	346		473.4	191.0
Void surface		281	721.2	237.9
				
With 2MeCN				
Void volume	0		211.6	0.3
Void surface		0	716.4	3.2

**Table 3:** Selected geometric parameters (Å, °)

Fe1—N1	2.127 (3)	Fe1—N4	2.147 (3)
Fe1—N2	2.141 (3)	Fe1—N101	2.169 (3)
Fe1—N3	2.142 (3)	Fe1—N201	2.185 (3)
			
N1—Fe1—N2	175.79 (12)	N101—Fe1—N201	98.60 (11)
N3—Fe1—N101	174.68 (12)	C106—N101—Fe1	162.4 (3)
N4—Fe1—N101	88.04 (12)	C206—N201—Fe1	161.2 (3)

**Table 4:** Hydrogen-bond geometry (Å, °)

*D*—H⋯*A*	*D*—H	H⋯*A*	*D*⋯*A*	*D*—H⋯*A*
C12—H12*A*⋯N5*A* ^viii^	0.98	2.58	3.454 (7)	149
C12—H12*B*⋯N103^viii^	0.98	2.54	3.443 (6)	154
C12—H12*C*⋯N204^vii^	0.98	2.56	3.469 (6)	154
C22—H22*A*⋯N5*A* ^ix^	0.98	2.39	3.308 (7)	156
C22—H22*A*⋯N6*B* ^iv^	0.98	2.61	3.41 (3)	138
C22—H22*B*⋯N204^iii^	0.98	2.52	3.407 (5)	150
C22—H22*C*⋯N103^ix^	0.98	2.52	3.424 (6)	154
C52—H52*B*⋯N102	0.98	2.51	3.492 (8)	176

Symmetry codes: (iii) x, y, z-1; (iv) -x+1, -y+1, -z+1; (vii) x, y-1, z; (viii) x, y, z+1; (ix) x, y+1, z.
