# Supplementary material for: Coordination chemistry of polynitriles. Part 9. Deca­cyano­ferrocene revisited: crystal and mol­ecular structure of cis-[{C5(CN)5}2(MeCN)4Fe]
Source: Acta Crystallogr C Struct Chem. 2022 Jan 21;78(Pt 2):94–100. doi: 10.1107/S2053229622000365 (PMC8815677; doi:10.1107/S2053229622000365)
Supplement: Supplementary file 3 [file c-78-00094-sup3.pdf]

Acta Cryst C
STRUCTURAL CHEMISTRY

Volume 78 (2022)

Supporting information for article:

Coordination chemistry of polynitriles. Part 9. Decacyanoferrocene revisited. Crystal and molecular structure of *cis*-[{$C_5(CN)_5$}$_2$($MeCN$)$_4$Fe]

Karlheinz Sünkel and Tobias Blockhaus

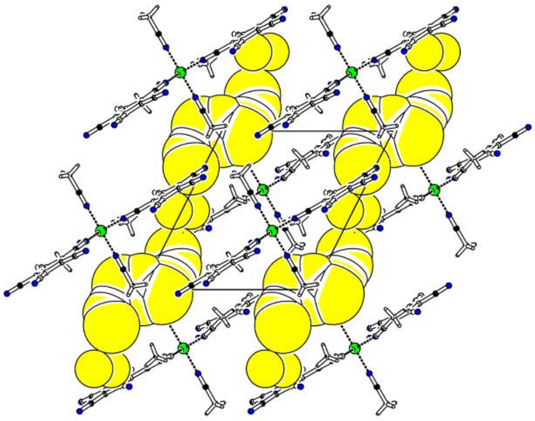

Figure S1 Platon cavity plot.

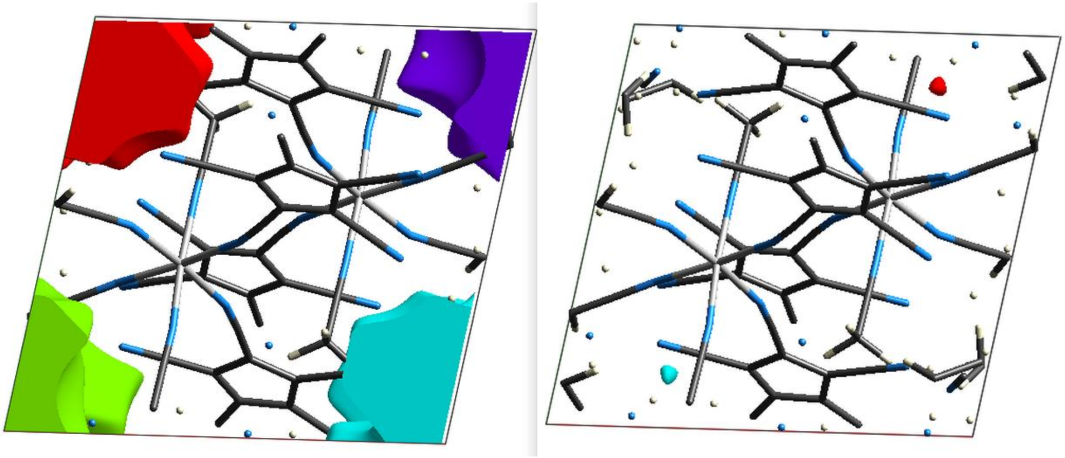

Figure S2 Void plots (0.0003 au Isosurface) for the structure solution without the lattice MeCN (left) and including the 2 MeCN lattice solvents (right). The surface area range from 65.8 (light blue) to 51.3 (violet) Å^2 in the left and both (light blue and red) 1.6 Å^2.

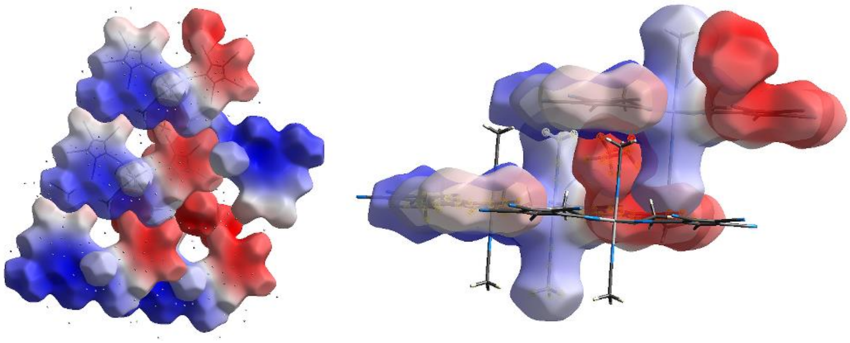

Figure S3 Top and side view of several "cloned" Hirshfeld surfaces displaying the electrostatic potentials and their complimentarity in neighbouring molecules.

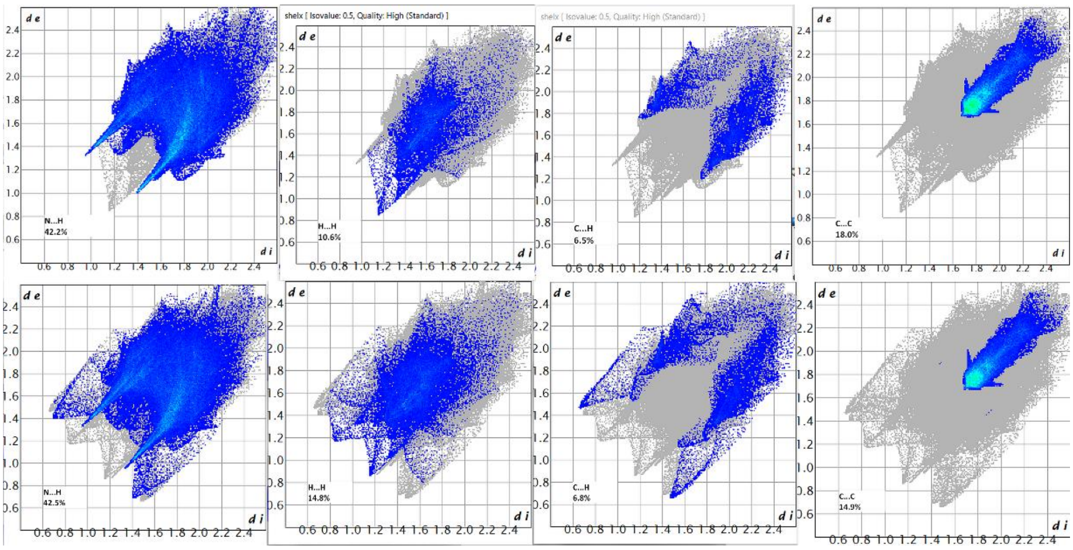

Figure S4 Individual Fingerprint plots corresponding to Figures 8a (top row) and 8b (bottom row).

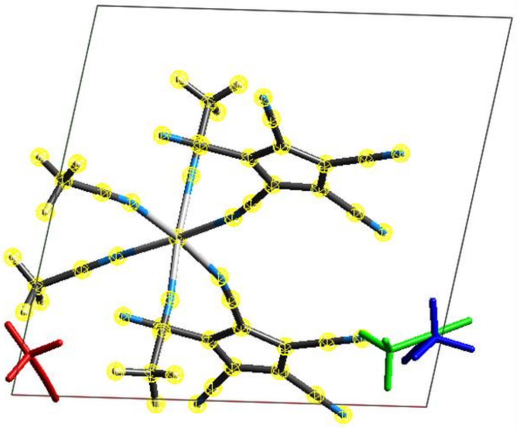

Information

Crystal | Atoms | Surface | Energies

Interaction Energies (kJ/mol)
R is the distance between molecular centroids (mean atomic position) in Å.

Total energies, only reported for two benchmarked energy models, are the sum of the four energy components, scaled appropriately (see the scale factor table below)

	N	Symop	R	Electron Density	E_ele	E_pol	E_dis	E_rep	E_tot
(red)	1	-	9.86	HF/3-21G	5.4	-2.9	-6.0	6.9	3.8
(green)	1	-	7.29	HF/3-21G	0.0	0.0	0.0	0.0	0.0
(blue)	1	-	7.24	HF/3-21G	0.0	-2.5	0.0	0.0	-1.6

Scale factors for benchmarked energy models
See Mackenzie et al. IUCrJ (2017)

Energy Model	k_ele	k_pol	k_disp	k_rep
CE-HF ... HF/3-21G electron densities	1.019	0.651	0.901	0.811
CE-B3LYP ... B3LYP/6-31G(d,p) electron densities	1.057	0.740	0.871	0.618

Figure S5 Interaction energies (HF/3-21G) between a single Fe complex and the two lattice MeCN molecules

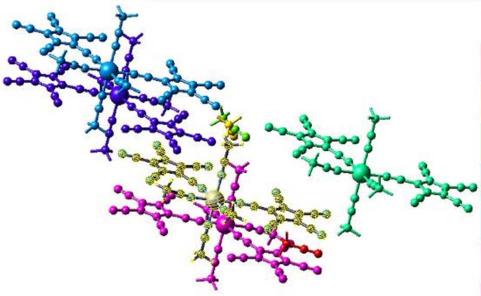

	N	Symop	R	E_ele	E_pol	E_dis	E_rep	E_tot
(red)	1	-	9.86	5.4	-2.9	-6.0	6.9	3.8
(yellow)	1	-	7.29	0.0	0.0	0.0	0.0	0.0
(green)	1	-	7.24	0.0	-2.5	0.0	0.0	-1.6
(teal)	1	x, y, z	12.87	-36.3	-11.8	-11.2	0.0	-54.8
(light blue)	1	x, y, z	13.09	-36.1	-11.8	-11.2	0.0	-54.5
(purple)	0	-x, -y, -z	11.68	-93.0	-26.0	-81.1	37.6	-154.3
(magenta)	1	-x, -y, -z	3.84	-46.3	-56.6	-188.6	98.5	-174.0

Figure S6 Interaction energies (HF/3-21G) between the asymmetric unit and four close neighbours.
